# Imaging Alzheimer's genetic risk using diffusion MRI: A systematic review

**DOI:** 10.1016/j.nicl.2020.102359

**Published:** 2020-07-22

**Authors:** Judith R. Harrison, Sanchita Bhatia, Zhao Xuan Tan, Anastasia Mirza-Davies, Hannah Benkert, Chantal M.W. Tax, Derek K. Jones

**Affiliations:** a Cardiff University Brain Research Imaging Centre (CUBRIC), Maindy Road, Cardiff CF24 4HQ, UK; b Cardiff University School of Medicine, University Hospital of Wales, Heath Park, Cardiff CF14 4XN, UK; c Mary MacKillop Institute for Health Research, Australian Catholic University, Melbourne, VIC, Australia

**Keywords:** Alzheimer's Disease, Magnetic Resonance Imaging, Diffusion Tensor Imaging, Apolipoproteins E, Presenilin-1, Presenilin-2, Multifactorial Inheritance

## Abstract

Diffusion magnetic resonance imaging (dMRI) is an imaging technique which probes the random motion of water molecules in tissues and has been widely applied to investigate changes in white matter microstructure in Alzheimer’s Disease. This paper aims to systematically review studies that examined the effect of Alzheimer’s risk genes on white matter microstructure. We assimilated findings from 37 studies and reviewed their diffusion pre-processing and analysis methods. Most studies estimate the diffusion tensor (DT) and compare derived quantitative measures such as fractional anisotropy and mean diffusivity between groups. Those with increased AD genetic risk are associated with reduced anisotropy and increased diffusivity across the brain, most notably the temporal and frontal lobes, cingulum and corpus callosum. Structural abnormalities are most evident amongst those with established Alzheimer’s Disease. Recent studies employ signal representations and analysis frameworks beyond DT MRI but show that dMRI overall lacks specificity to disease pathology. However, as the field advances, these techniques may prove useful in pre-symptomatic diagnosis or staging of Alzheimer’s disease.

## Highlights

• dMRI uses the movement of water molecules to quantify white matter microstructure.
• dMRI has demonstrated changes in white matter microstructure in Alzheimer’s Disease.
• We provide a systematic review of 37 dMRI studies of Alzheimer’s genetic risk.
• Alzheimer’s risk genes were linked to decreased anisotropy and greater diffusivity.
• Signal changes are non-specific, and the field faces several methodological challenges.

## 1 Introduction

Alzheimer’s Disease (AD) is a progressive neurodegenerative disorder affecting older adults. It is characterised by amyloid plaques, hyperphosphorylated tau and atrophy (Braak and Braak, 1995). Histopathological studies have also identified AD pathology in white matter (Englund, Brun and Alling, 1988). In recent years, diffusion Magnetic Resonance Imaging (dMRI) has been used to examine white matter microstructure in AD and to study the effect of AD genetic risk on white matter microstructure.

### 1.1 Alzheimer’s disease genetic risk

Sporadic AD, often called late-onset AD, is the most common form of dementia, affecting 1 in ten people over the age of 65 (Alzheimer’s Association, 2019). The heritability of sporadic AD is estimated to be between 58 and 79% (Gatz et al., 2006). The largest genome-wide association study (GWAS) of clinically confirmed AD has identified 25 loci that are associated with increased risk for sporadic AD (Kunkle et al., 2019). These are common genetic variants, known as single nucleotide polymorphisms (SNPs). The largest of these genetic risks are SNPs in the Apolipoprotein E (APOE) region. Carriers of two copies of the APOE Epsilon 4 (APOE E4) allele have an eight-fold increase in risk compared to non-carriers (Corder et al., 1993). In comparison to APOE, other common risk loci have only a modest effect on disease risk. However, their combined effect can be studied using polygenic risk scores. These are calculated from the weighted sum of weighted allelic dosages across the genome, and have proven particularly effective in predicting AD (Escott-Price et al., 2015). In addition to common genetic risk captured by GWAS, advances in sequencing techniques have assessed entire exomes and genomes, identifying rare mutations with moderate-to-strong effects. For example, TREM2 is a variant that encodes the trigger receptor expressed on myeloid cells 2 (Guerreiro et al., 2013). Other novel variants are involved in immune response and transcriptional regulation (Bis et al., 2018).

Whilst sporadic AD occasionally occurs in people under the age of 65, autosomal-dominant AD is characterised by an early clinical onset. In contrast to sporadic AD, autosomal-dominant AD is rare (Alzheimer’s Association, 2019). It is caused by mutations either in the amyloid precursor protein (APP) gene, or in presenilin 1 and 2 (PS1 and PS2) that are involved in cleaving amyloid β and APP. The disease onset is often predictable, depending on the specific mutation (Tanzi, 2012).

### 1.2 Diffusion MRI

dMRI is a non-invasive imaging method that probes the movement of water molecules to assess the microstructural configuration of white matter tracts (Jones, 2011, Winston, 2012). dMRI measures indicate how readily water molecules can diffuse in and around structures such as white matter fibres or cell bodies (Stejskal and Tanner, 1965, Bihan, 1995, Strijkers et al., 2011, Johansen-Berg and Behrens, 2013). In white matter, the rate of diffusion is modulated by multiple microstructural features including axon diameter, axon density and myelination (Jones, 2011). In highly ordered white matter, the rate of diffusion is anisotropic, i.e., it is strongly dependent on the direction in which it is measured. The commonly index of anisotropy is the fractional anisotropy (FA) introduced by Basser and Pierpaoli (Basser and Pierpaoli, 1996). An FA of 0 indicates that the rate of diffusion is the same in all directions (isotropic diffusion), and 1 represents the extreme case where diffusion can only occur along one axis (anisotropic diffusion) (Beaulieu and Allen, 1994, Pierpaoli and Basser, 1996, Beaulieu, 2009, Winston, 2012). Clinical studies often employ this as a measure of tissue integrity (Thomason and Thompson, 2011), although at best this interpretation is an oversimplification (Jones, Knösche and Turner, 2013). Another widely used metric is mean diffusivity (MD), which represents the orientationally-averaged rate of diffusion. Additional commonly used metrics from DTI are the ‘longitudinal diffusivity’ (LD) and ‘radial diffusivity’ (RD), which in turn represent the highest and lowest rates of diffusion. In the case of perfectly aligned axonal bundles, these would represent diffusivity parallel and perpendicular to the main axis of the bundle, respectively. However, given the ubiquity of multiple fibre populations within an image voxel, this interpretation carries some risk (see: Wheeler-Kingshott and Cercignani, 2009) but also see (Wheeler-Kingshott et al., 2012)). Collectively, FA, MD, LD and RD can help to characterise changes in diffusion resulting from differences in white matter microstructure.

### 1.3 Structural changes observed in Alzheimer’s disease

Conventional MRI measures of atrophy, such as Voxel-Based Morphometry (VBM), are established markers for AD diagnosis and measurement of progression (Frisoni et al., 2010). More recently, dMRI has allowed the exploration of AD white matter microstructure, finding widespread changes. A *meta*-analysis of 41 studies found reduced FA and increased MD in AD brains compared to controls. Differences were marked in frontal and temporal lobes, and the posterior cingulum, corpus callosum, superior longitudinal fasiculi and uncinate fasiculi (Sexton et al., 2011). Late-myelinating tracts may be affected primarily by AD neurodegeneration (Benitez et al., 2014). Longitudinal studies suggest that the pattern of decreased FA and increased MD becomes more distinct as the disease progresses (Mayo et al., 2017). Changes in the parahippocampal cingulum have been shown to discriminate between AD and healthy controls (Mayo et al., 2017). Diffusion measurements in the fornix are another possible biomarker (Ringman et al., 2007). Perea and colleagues found that AD preferentially degraded the crus and body of the fornix. The diffusion differences remained after controlling for fornix volume (Perea et al., 2018).

Mild Cognitive Impairment (MCI) describes a degree of cognitive problems that do not affect day-to-day living, and are considered to be an AD prodrome (Petersen and Morris, 2005). A *meta*-analysis of 41 studies found that compared to healthy controls, patients with MCI had lower FA in all white matter areas except parietal and occipital regions, and higher MD except in occipital and frontal regions (Sexton et al., 2011). More recently, whole brain white matter histogram analysis found that RD, LD and MD were able to discriminate between AD and controls and between MCI and controls in the ADNI cohort. LD appeared to be the most sensitive marker (Giulietti et al., 2018).

dMRI metrics in the fornix are markers of cognitive problems, and can distinguish MCI from AD (Egli et al., 2015, Tang et al., 2017). The volume of the body of the fornix and LD in the fornix are correlated with decline from normal cognition (Fletcher et al., 2013). Reduced FA in the fornix can predict conversion both from healthy cognition to MCI and from MCI to AD with high specificity and > 90% accuracy (Mielke et al., 2012, Oishi et al., 2012). Reduced FA and increased MD in the fornix might even precede hippocampal atrophy (Zhuang et al., 2012).

### 1.4 Current review

This systematic review aimed to collate studies applying diffusion MRI techniques to investigate genetic risk for AD. In our narrative synthesis, our goal is to assess the evidence for manifestations of Alzheimer’s genetic risk in white matter microstructure. We also aim to review the studies in terms of their study design and diffusion methodology, including pre-processing and analysis.

## 2 Methods

We conducted this systematic review in accordance with PRISMA guidelines (Moher et al., 2009).

### 2.1 Study selection

Initially, we defined our search terms (listed in Table 1, Supplementary Materials). We searched the literature using MEDLINE, PSYCHINFO and EMBASE from January 2000-July 2019. We hand-searched the reference lists of related articles.

Inclusion criteria:

• Case-control, cross-sectional or longitudinal studies
• Genotyped participants
• Imaged with dMRI sequences
• Associations reported between AD risk genes/SNPs and measures derived from dMRI

Exclusion criteria:

• Publications in non-English language journals
• Conference proceedings
• Studies of non-Alzheimer’s dementia or unspecified dementia
• Studies using family history and genotype as a composite variable
• Studies using MRI but not including dMRI
• Studies investigating genes/SNPs that are not associated with AD risk
• Studies that co-vary for AD genes (e.g. APOE) but that do not report associations with AD risk genes/SNPs

### 2.2 Article selection

The articles included in this review are all English language original research papers. Study designs included case-control, cross-sectional and longitudinal studies. The primary search was conducted by SB. Five reviewers (SB, HB, AMD, ZXT, JH) all independently selected studies based on the eligibility criteria. Disagreements were resolved by consensus.

### 2.3 Data extraction

Reviewers (SB, HB, AMD, ZXT, JH) extracted information from papers independently. Data were extracted from each study in duplicate to ensure consistency. Key data included: study design and number of participants the AD genetic risk measured; MRI acquisition parameters; dMRI pre-processing; dMRI analysis techniques; reported findings. A complete list of the data extracted can be found in Supplementary Materials Table 2.

### 2.4 Quality assessment

The quality of each included study was assessed independently by two reviewers using the appropriate version of the Newcastle-Ottawa Scale (NOS) (Stang, 2010) for the study design (case/control, cross-sectional or cohort study). The NOS assesses the quality of non-randomized studies in three main areas: the selection of study groups; the comparability of the groups; and the ascertainment of the exposure or outcome of interest. This tool was chosen because of the type of studies included. A consensus meeting between all reviewers established a manual to ensure this was applied consistently. The assessment tool was adapted to fit the included studies, where the exposure was defined as genetic risk, and important covariates were age, sex and APOE e4 status. A point was awarded in each category.

## 3 Results

### 3.1 Search results

**Fig. 1:**
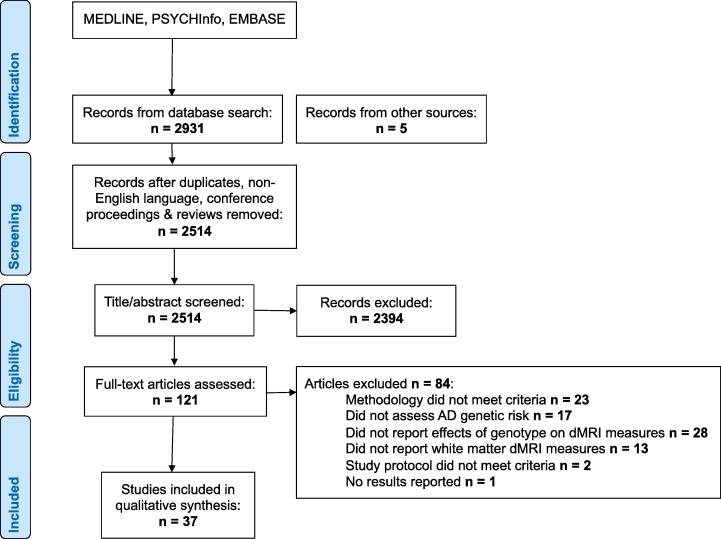
PRISMA flow chart.

We identified 2931 articles in our initial search (see PRISMA flow diagram in Fig. 1). We excluded duplicates, non-English language, non-human studies and conference proceedings. 2514 articles were screened based on their titles and abstracts and a further 2394 were excluded. The reviewers (SB, HB, AMD, ZXT, JH) reviewed the full text of 120 articles and applied the inclusion criteria. 32 studies met the criteria for inclusion. A further 4 studies were identified through hand-searches of reference lists.

### 3.2 Study characteristics: Study design, sample, Alzheimer’s genetic risks

The majority of the studies were case/control design, although some were cross-sectional (Foley et al., 2016) and some longitudinal cohort studies (Lyall et al., 2014). Some studies were conducted using the same cohorts: three used data from the Beijing Aging Brain Rejuvenation Initiative (BABRI); two used the Wisconsin Registry for Alzheimer’s Prevention (WRAP); two used the European Diffusion Tensor Imaging Study on Dementia (EDSA) and the DZNE database, Rostock, Germany. Only one article reported data from the Alzheimer’s Disease Neuroimaging Initiative (ADNI). Most studies included participants who were pre-symptomatic. Only ten included those with established AD or MCI.

### 3.3 Genotypes

Two approaches were used to assess genetic risk for AD. Most studies tested participants for specific mutations (APP, PS1/2 mutations or APOE alleles). One study used an array to genotype participants and calculate polygenic risk scores based on sporadic AD GWAS (Foley et al., 2016).

### 3.4 dMRI pre-processing and analysis methods

Prior to modelling or statistical analysis, it is essential to pre-process the dMRI data, correcting for artefacts, motion and eddy-current induced distortions (Jones, Knösche and Turner, 2013). Once pre-processed, different approaches can be applied to represent the dMRI signal. Beyond the diffusion tensor framework, two common ways to represent the orientation dependence of the signal in dMRI are the diffusion orientation density function (dODF) (Wedeen et al., 2005) and the fibre orientation density function (fODF) (Dell’Acqua et al., 2019). The diffusion ODF is a spherical function which characterises the probability of diffusion along a unit direction. On the other hand, the fODF is a function that characterises the probability of finding a fibre oriented along a particular axis (Jones et al., 2013).

An additional method, known as neurite orientation dispersion and density imaging (NODDI), aims to provide more specific microstructural information (Zhang et al., 2012). NODDI assumes there are three biophysical compartments in white matter, intra-cellular, extra-cellular and cerebrospinal fluid, in a single voxel. By imposing constraints on some of the parameters that describe these compartments, NODDI aims to estimate proxies of intracellular volume fraction (IVF), neurite density index (NDI), orientational dispersion index (NDI) and increased free isotropic water fraction (FISO) (Zhang et al., 2012).

Quantitative dMRI measures, such as FA, MD, RD and LD (all derived from the diffusion tensor), can be analysed using tractography or whole-brain voxel-wise analysis. Tractography involves reconstructing the trajectory of fibres and connection patterns, using either the principal eigenvector of the diffusion tensor, or peaks in the dODF or fODF, within successive adjacent voxels (Tournier et al., 2011, Jones et al., 2013). These local orientations are used to infer total fibre trajectories (Jeurissen et al., 2019). Commonly used methods include deterministic and probabilistic tractography. In deterministic tracking, a path is propagated along local maxima of the ODF (or, in the case of diffusion tensor imaging, along the principal eigenvector). However, imaging noise and artifacts can make estimates of local maxima imprecise and adds some local orientational uncertainty. Probabilistic tractography techniques illustrate these uncertainties by assigning an uncertainty, or conversely, a probability to the orientational estimates. As such, each local maximum in an ODF can generate a collection of possible trajectories (Jeurissen et al., 2019).

A tractography-based region-of-interest (ROI) approach allows the researcher to define ‘seeds’ to begin fibre tracking, or to define ‘way-points’ that prescribe regions through which a reconstructed tract must pass in order to be retained for analysis (Conturo et al., 1999). These can be drawn manually or automatically. Alternatively, whole-brain tractography places seeds throughout the whole brain (Soares et al., 2013), again using ‘way-point’ ROIs to filter out target pathways. In tractography, each tract is segmented in the native of the individual (rather than requiring that the individual’s data are co-registered to some standardised template space, providing a representation of tract anatomy for each individual (Bastin et al., 2013)). It is important to recognise that the reconstructed tracts do not represent nerve fibres or fibre bundles directly. Rather, they represent pathways or trajectories through the signal, and we infer that the nerve fibres run approximately in parallel. These pathways can be translated into qualitative information, e.g., on the tract shape, and into quantitative information, as measures averaged along the tract (Jones and Pierpaoli, 2005) or in assessing the extent of connections between brain regions (Kaden, Knösche and Anwander, 2007).

Whole brain voxel-based techniques, such as Tract Based Spatial Statistics (TBSS) (Smith et al., 2006, Smith et al., 2007) or Voxel-Based Analysis (VBA) (Büchel et al., 2004, Van Hecke et al., 2009), are an alternative approach to tractography. They typically involve the nonlinear registration of quantitative diffusion tensor imaging maps, (e.g. FA), from each individual to a standard template space. The aligned FA images are then averaged, and a skeletonised mean FA structure is created. Thresholds are applied to suppress areas of low mean FA or high inter-subject variation. Each subject's FA image is then projected onto the skeleton, and voxel-wise statistics can be carried out across subjects. For comprehensive descriptions of these different dMRI methods and possible pitfalls, please see (Smith et al., 2006, Jones et al., 2013, Soares et al., 2013).

Inter-regional connectivity can be assessed by constructing networks of the human brain using diffusion signals and tractography (Yeh et al., 2020). The resultant networks can be characterised using graph theoretical approaches. Graph theory is a mathematical framework for representing complex networks. The brain can be illustrated using nodes, representing regions or voxels, and edges, representing connections between nodes (Bullmore and Sporns, 2009). A number of network metrics can be produced such as small-world and network efficiency. Please see (Boccaletti et al., 2006) for a detailed summary of graph theory.

The studies that met our inclusion criteria used a range of dMRI analysis methods. 15 used TBSS, seven used a tractography-based ROI approach, eight used VBA, three combined TBSS and VBA, one combined TBSS and ROI, and three calculated structural connectivity matrices.

### 3.5 Studies of white matter structure and APOE status

The majority of the papers which met our inclusion criteria explored the effects of APOE (27 articles). Most used a case-control design, although some were longitudinal studies. There was a wide range of sample sizes (N range = 14–885). The literature predominantly examined samples of cognitively healthy older adults (age > 60). Five studies included participants with diagnoses of AD or MCI (Bagepally et al., 2012a, Bagepally et al., 2012b, Kljajevic et al., 2014, Wai et al., 2014, Ma et al., 2017, Slattery et al., 2017). Studies of younger age groups included adolescents (Dell’Acqua et al., 2015), adults in their 20′s (Heise et al., 2011, O’Dwyer et al., 2012, Dowell et al., 2013), 40′s and 50′s (Westlye et al., 2012, Operto et al., 2018). Some studies were able to compare groups with different combinations of APOE alleles (Lyall et al., 2014), although most simply compared APOE E4 carriers (homozygotes and heterozygotes) to those without an E4 allele. Diffusion methodology included: TBSS (12 studies); tractography-based ROI (6 studies); VBA (4 studies); TBSS and VBA (3 studies); TBSS and ROI (1 study); structural connectivity (3 studies). Table 1 provides a summary of studies exploring white matter metrics and APOE genotype.

**Table 1:** Summary of sample characteristics, methodology and main findings for studies of APOE.

Study	N (E4 carriers; non-carriers)	Age (SD)	dMRI Method	Field Strength (T)	B value (s/mm2)	Acquisition Voxel Size (mm)	N Directions	NEX	Regions of Interest	Results
Adluru et al, 2014	343 (123; 220)	61.03 (6.72)	ROI	3	0, 1300	2.5 × 2.5 × 2.5	40	1	Fornix, splenium & genu of corpus callosum, cingulum, uncinate, superior longitudinal fasiculus.	• Older (>65) carriers vs. non-carriers: ↑MD in SLF & cingulum bundle
Bagepally et al, 2012	32 (19; 13)	69.3 (5.7)	TBSS	3	0, 1000	Not reported	32	1	Whole brain	• AD carriers vs. AD non-carriers: ↓ FA in left medial temporal areas, parahippocampal cingulum, bilateral intracalcarine sulcus, precuneus, lingual area • Healthy carriers vs. healthy non-carriers: ↓ FA bilateral medial temporal areas, scattered regions in frontal & parietal lobes & cerebellum
Bendlin et al, 2010	136 (56; 80)	69.2 (10.2)	VBA	3	0	2 × 2 × 3	12	1	Whole brain	• Carriers vs. non-carriers: no significant differences
Brown et al, 2011	55 (25; 30)	62.3 (9.0)	Structural connectivity	3	800 or 1000	2.5 × 2.5 × 2.5	30	1	Global & regional connectivity	• Carriers vs. non-carriers: age-related loss of mean local interconnectivity, & regional local interconnectivity decreases in the precuneus, medial orbitofrontal cortex, & lateral parietal cortex.
Cai et al, 2017	309 (116; 193)	73.9 (4.6)	VBA	3	Not reported	1.02 × 1.02 × 1.02	Not reported	1	Superior corona radiata, genu of corpus callosum	• Carriers vs. non-carriers: ↑ MD in superior corona radiata, genu of corpus callosum. No significant associations with FA or RD
Cavedo et al, 2017	74 (31; 43) across 4 centres	68.9 (6.9)	TBSS, VBA	1.5 or 3	0, 1000 or 0, 800	2 × 2 × 2 or 1 × 1 × 2.4	12, 15, 20 or 60	1	Whole brain	• Carriers vs. non-carriers: ↓ FA globally, and in genu, body & splenium of corpus callosum, internal capsule, external capsule, inferior fronto-occipital & inferior longitudinal fasciculi, cingulum (left & right). ↑ MD in right hemisphere, in genu of corpus callosum, right internal capsule, right corona radiate, right superior longitudinal fasiculus. ↑ RD globally, & bilaterally in genu & splenium of corpus callosum, internal capsule, inferior fronto-occipital & inferior longitudinal fasciculi, cingulum, external capsule
Chen et al, 2016	75 (35; 40)	65.8 (7.5)	Structural connectivity	3	0, 1000	Not reported	30	3	Whole brain structural network	• Carriers vs. controls: lower global efficiency, no significant differences in local efficiency. Decreased nodal efficiency in left anterior cingulate, left paracingulate gyrus, right dorsolateral superior frontal gyrus, left inferior occipital gyrus • Case/control prediction: ROC AUC for global efficiency 0.74; decreasing region 0.81
Dell'Acqua et al, 2015	575 (119; 374)	14.4 (0.5)	TBSS	3	0, 1300	2.4 × 2.4 × 2.4	60	1	Whole brain	• Carriers vs. non-carriers: no significant differences
Dowell et al, 2013	41 (20; 21)	20.0 (2.0)	TBSS	1.5	0, 1000	2.5 × 2.5 × 2.5	30	1	Whole brain	• Carriers vs. controls: ↑ RD in carriers, partidcularly in inferior longitudinal fasciculus. No significant differences in FA or MD.
Heise et al, 2010	71 (33; 38)	Young cohort 28.6 (4.2); Older cohort: 64.9 (7.2)	TBSS		1000	1.1 × 0.9 × 3		1	Whole brain	• Young carriers vs. non-carriers: ↓ FA in cingulum, corona radiata, corpus callosum, external capsule, internal capsule, superior longitudinal fasciculus • Older carriers vs. non-carriers: ↑ MD in cingulum, corona radiata, corpus callosum, external capsule, internal capsule, superior longitudinal fasciculus • All carriers vs. non-carriers: ↓ FA in cingulum, corona radiata, corpus callosum, external capsule, internal capsule, superior longitudinal fasciculus
Honea et al, 2009	53 (14; 39)	73.4 (6.3)	TBSS	3	0, 1000	1 × 1 × 1	12	1	Whole brain	• Carriers vs. non-carriers: no significant differences
Kljajevic et al, 2014	126 (63; 63) across 5 centres	67.7 (5.9)	VBA	1.5 + 3	0, 800 or 0, 1000	2 × 2 × 2, 2 × 2 × 2.5 or 2 × 2 × 3	6 or 20	1	Whole brain	• Healthy carriers vs. healthy non-carriers: ↑ MD in left lentiform nucleus • AD carriers & AD non-carriers: ↓ FA in middle frontal areas, insular white matter, superior temporal areas
Laukka et al, 2015	89 (23; 66)	81.4 (3.0)	TBSS	1.5	600	1 × 1 × 1	6	1	Cingulate gyrus part of cingulum, parahippocampal cingulum, corticospinal tract, forceps major & minor, inferior fronto-occipital fasciculus, superior longitudinal fasciculus	• Carriers vs. non-carriers: ↓ FA in forceps major, ↑ MD in corticospinal tract
Lyall et al, 2014	645 (187; 423)	72.7 (0.7)	ROI	1.5	0, 1000	1.8 × 1.8 × 1.8	64	1	Genu & splenium of corpus callosum, bilateral anterior thalamic radiations, ventral & rostral cingulum bundles, & arcuate, uncinate, & inferior longitudinal fasciculi.	• Carriers vs. non-carriers: ↓ FA in right ventral cingulum & left inferior longitudinal fasciculus
Ma et al, 2017	885 (145; 729)	65.3 (7.4)	Structural connectivity	3 T	1000	Not reported	30	1	Whole brain structural network	•Healthy carriers vs healthy non-carriers: ↑ clustering coefficient & local efficiency • MCI carriers vs. non-carriers: ↓ clustering coefficient & local efficiency • All carriers vs. non-carriers: ↓ nodal efficiency in: inferior frontal gyrus, orbital part; left superior frontal gyrus, orbital part; left middle occipital gyrus. ↑ nodal efficiency in: left cuneus; left inferior parietal but supramarginal and angular gyri
Newlander et al, 2014	14 (7; 7)	72.7 (6.1)	VBA	1.5	0, 800	1 × 1 × 1	12	1	Whole brain	• Carriers vs. non-carriers: ↓ FA in genu of corpus callosum & brain stem
Nierenberg et al, 2005	29 (14; 15)	67.1 (6.5)	ROI	1.5	0, 1000	Not reported	20	1	Parahippocampal cingulum	• Carriers vs. non-carriers: ↓ FA & ↑ RD in parahippocampal cingulum
Nyberg et al, 2014	273 (69; 204)	67.01 (8.0)	TBSS, VBA	3	1000	0.98 × 0.98 × 2	32	1	Whole brain	• Carriers vs. controls: no significant difference for whole brain metrics or specific subregion metrics in TBSS. ↓ FA in five anterior and posterior midline regions on VBA
O'Dwyer et al, 2012	44 (22; 22)	26.7 (4.0)	TBSS	3	1000	2 × 2 × 2	60	1	Whole brain	• Carrier vs. non-carrier: no significant differences in diffusion indices • Carrier/non-carrier prediction accuracy: sensitivity & specificity range 93–100% using a feature selection algorithm, support vector machines & FA data
Operto et al, 2018	532 (275; 257)	58.1 (7.5)	TBSS	3	0, 1000	2 × 2 × 2	64	1	Whole brain	• Carriers vs. non-carriers: ↑ MD, RD & LD in corona radiata, superior longitudinal fasciculus, inferior longitudinal fasciculus, inferior fronto-occipital fasciculus, corticospinal tract
Persson et al, 2006	60 (30; 30)	66.3 (7.7)	ROI	Not reported	1000	Not reported	6	1	Whole brain analysis; genu, splenium, body of corpus callosum	• Carriers vs. non-carriers: ↓ FA in occipito-frontal fasciculus, body of corpus callosum
Ryan et al, 2011	126 (36; 88)	69.2 (10.2)	ROI	3	0, 1000	2.6 × 2.6 × 2.6	25	2	Frontal white matter, lateral parietal white matter, centrum semiovale, genu of the corpus callosum, splenium of the corpus callosum, temporal stem white matter.	• Carriers vs. non-carriers: no significant difference in LD, ↓ FA in frontal white matter & splenium
Salminen et al, 2013	64 (23; 41)	61.75 (7.6)	ROI	3	0, 996	2 × 2 × 2	31	1	Left uncinate fasciculus, right uncinate fasciculus, temporal lobe	• Carriers vs. controls: ↓ fibre bundle length in left uncinate fasciculus
Slattery et al, 2017	37 (22; 15)	61.7 (5.0)	TBSS, ROI	3	1000	2.5 × 2.5 × 2.5	64	1	Quadrants (anterior, posterior, left & right)	• AD carriers vs. AD non-carriers: ↓ FA in splenium of corpus callosum & anterior corona radiata. ↑ RD in white-matter projections from frontal lobes. More widespread ↓ NDI in parieto-occipital white-matter projections. ↑ FISO in corpus callosum • Healthy carriers vs. non-carriers: no significant differences
Smith et al, 2016	88 (34; 53)	74.1 (4.5)	TBSS	3	0, 1000	1 × 1 × 1	25	2	Longitudinal fasciculus, sagittal stratum, uncinate fasciculus, cingulate gyrus, parahippocampal cingulumfornix, body, genum & splenium of corpus callosum, internal capsule, corona radiata	• Carriers vs. non-carriers: no significant effects on FA, LD or MD. ↑ RD in right anterior internal capsule, bilateral posterior corona radiata, left superior corona radiata.
Wai et al, 2014	120 (22; 98)	68.9 (7.7)	TBSS	3	1000	2 × 2 × 2	64	1	Whole brain	• Amnestic MCI carriers vs. controls: ↓ FA & ↑ MD
Wang et al, 2015	241 (73; 126)	72.0 (9.0)	TBSS	1.5	0, 600	1 × 1 × 1	6	1	Whole brain	• Carriers vs. non-carriers: no significant differences
Westlye et al, 2012	203 (60; 143)	47.6 (14.9)	TBSS	1.5	1000	2 × 2 × 2	60	1	Whole brain	• Carriers vs. non-carriers: increased RD brainstem, basal temporal lobe, internal capsule, anterior parts of the corpus callosum, forceps minor, superior longitudinal fasciculus, occipital & corticospinal pathways.
Zhang et al, 2015	75 (35; 40)	65.9 (7.5)	TBSS, VBA	3	0, 1000	2 × 2 × 2	30	1	Genu, body, splenium of corpus callosum, anterior & posterior corona radiate, fornix, cerebral peduncles, parahippocampal cingulum	• Carriers vs. non-carriers: ↓ FA & ↑ MD in genu & body or corpus callosum, anterior & posterior corona radiate bilaterally with TBSS. ↓ FA in genu and body of corpus callosum & right fornix stria terminalis , ↑ MD in genu & splenium of corpus callosum, right cerebral penduncle & right parahippocampal cingulum with VBA

Please note that we report findings from the most rigorous analyses conducted by studies, including models controlling for multiple comparisons. When not otherwise reported, NEX was assumed to be 1. Acronyms: dMRI = diffusion Magnetic Resonance Imaging; E4 = APOE Epsilon 4; TBSS = Tract-Based Spatial Statistics; VBA = Voxel-Based Analysis; ROI = Region of Interest; FA = Fractional Anisotropy; MD = Mean Diffusivity; RD = Radial Diffusivity; LD = Longitudinal Diffusivity; NDI = Neurite Dispersion Index; FISO = Free Isotropic Water Fraction; ROC = Receiver Operating Characteristic; AUC = Area Under Curve.

Five studies reported no significant differences in white matter microstructure between carriers and non-carriers (Honea et al., 2009, Bendlin et al., 2012, Nyberg and Salami, 2014, Dell’Acqua et al., 2015, Wang et al., 2015). All other studies reported some significant changes in diffusion metrics associated with *APOE*4. The pattern of alteration in affected tracts or regions was similar to studies of autosomal-dominant AD genes: reduced FA was commonly reported, often in tandem with increased MD, RD or LD. Reduced neurite density index (NDI) and increased free isotropic water fraction (FISO) are also reported. The white matter regions found to be associated with APOE status included: the genu (Newlander et al., 2014, Zhang et al., 2015, Cai et al., 2017, Cavedo et al., 2017), body (Persson et al., 2006, Zhang et al., 2015) and splenium (Ryan et al., 2011, Slattery et al., 2017) of the corpus callosum and the corpus callosum overall (Heise et al., 2011, Westlye et al., 2012, Cavedo et al., 2017); the parahippocampal cingulum (Nierenberg et al., 2005, Bagepally et al., 2012a, Bagepally et al., 2012b, Kljajevic et al., 2014, Zhang et al., 2015) and the cingulum overall (Adluru et al., 2014, Lyall et al., 2014, Cavedo et al., 2017); the intracalacrine sulcus (Bagepally et al., 2012a, Bagepally et al., 2012b, Westlye et al., 2012); the brain stem (Westlye et al., 2012, Newlander et al., 2014); the corona radiata (Heise et al., 2011, Smith et al., 2016, Cai et al., 2017, Cavedo et al., 2017, Slattery et al., 2017, Operto et al., 2018); the external capsule (Heise et al., 2011, Cavedo et al., 2017) and internal capsule (Heise et al., 2011, Westlye et al., 2012, Smith et al., 2016, Cavedo et al., 2017); the superior longitudinal fasciculus (Adluru et al., 2014, Lyall et al., 2014, Cavedo et al., 2017, Operto et al., 2018) and inferior longitudinal fasciculus (Dowell et al., 2013, Cavedo et al., 2017); the fronto-occipital fasiculus (Cavedo et al., 2017, Operto et al., 2018); the fornix (Zhang et al., 2015); the cerebral peducles (Zhang et al., 2015); the cortico-spinal tract (Laukka et al., 2015); the uncinate fasiculus (Salminen et al., 2013); the forceps major (Laukka et al., 2015) and forceps minor (Operto et al., 2018).

Three papers used measures of structural connectivity based on graph theory. Brown et al found that APOE E4 carriers had age-related loss of mean local interconnectivity and regional local interconnectivity in the precuneus, medial orbitofrontal cortex, and lateral parietal cortex (Brown et al., 2011). Ma et al studied participants with MCI and with normal cognition. They found that healthy APOE E4 carriers had increased clustering coefficient and local efficiency compared to healthy non-carriers. In those with MCI, carriers showed decreased clustering coefficient and local efficiency relative to MCI non-carriers. When all carriers were compared to all non-carriers, they showed decreased nodal efficiency in the inferior frontal gyrus, the left superior frontal gyrus, and the left middle occipital gyrus. Carriers also showed increased nodal efficiency in the left cuneus, the left inferior parietal, supramarginal and angular gyri (Ma et al., 2017). A further study reported that E4 carriers had lower global efficiency but no significant differences in local efficiency. Decreased nodal efficiency in left anterior cingulate, left paracingulate gyrus, right dorsolateral superior frontal gyrus, and left inferior occipital gyrus was reported in carriers relative to non-carriers. In addition, they used structural connectivity measures to predict AD with Receiver-Operator Curves (ROC). Using global efficiency, they produced an Area Under Curve (AUC) of 0.74. Using mean nodal efficiency of significant decreasing regions, this improved to 0.81 (Chen et al., 2015).

### 3.6 Studies of white matter and autosomal-dominant AD genes

Six studies explored white matter metrics in participants with autosomal-dominant AD genes: three studied PS1 carriers, two studied APP and PS1 carriers and one studied PS1, PS2 and APP carriers. All used a case/control design. They compared pre-symptomatic and symptomatic gene carriers to non-carriers. Sample sizes reflect the rarity of the genes (N range = 20–109, of which 10–64 were carriers). Three studies used VBA, three used TBSS.

Of the three studies of PS1 carriers, one study identified reduced MD and LD in the right cingulum among pre-symptomatic carriers (Ryan et al., 2013), and the other two studies reported no significant differences between pre-symptomatic PS1 and non-carriers (Parra et al., 2015, Sanchez-Valle et al., 2016). In symptomatic PS1 carriers, changes included: increased MD, RD and LD and reduced FA in all the fornix, cingulum and corpus callosum (Ryan et al., 2013); higher MD in the left inferolateral frontal white matter, right parahippocampal cingulum bundle, splenium left of the mid-line and genu symmetrically around the mid-line of the callosum (Parra et al., 2015); decreased FA in the genu and body of corpus callosum and corona radiata bilaterally and increased MD, LD, and RD in the splenium of corpus callosum relative to age (Sanchez-Valle et al., 2016).

Two studies with mixed cohorts of *PS1* or *APP* carriers reported a number of changes in pre-symptomatic carriers: reduced FA in the fornix and frontal white matter (Ringman et al. 2007); increased MD in the left inferior longitudinal fasciculus, left forceps major, left cingulum and bilateral superior longitudinal fasciculus (Li et al., 2015). In the same PS1/APP studies, symptomatic carriers showed: decreased mean FA across the whole brain, especially in the left frontal white matter, and right and left perforant paths (Ringman et al. 2007); increased MD in the inferior longitudinal fasciculus, forceps major, cingulum and bilateral superior longitudinal fasciculus (Li et al., 2015). The effects seen in the symptomatic APP/PS1 carriers were greater and more widespread than in pre-symptomatic carriers (Ringman et al., 2007, Li et al., 2015). Caballero et al studied a large mixed cohort of PS1/2 and APP carriers. They found increased MD in the forceps minor, forceps major and long projecting fibres 5–10 years before the estimated onset of symptoms (Caballero et al., 2018). See Table 2 for a summary of these studies.

**Table 2:** Summary of sample characteristics, methodology and main findings for studies of FAD genes.

Study	Gene	N (FAD carriers; non-carriers)	Mean Age (SD)	dMRI Method	Field Strength (T)	B value (s/mm2)	Acquisition Voxel Size (mm)	N Directions	NEX	Region of Interest	Results
Caballero et al, 2018	PS1, PS2, APP	109 (64; 45)	Symptomatic & pre-symptomatic 38.8 (10.6); Non-carriers 38.0 (11.2)	TBSS	3	0, 1000	0.9 × 0.9 × 5.0	64	1	Whole brain	• Symptomatic & pre-symptomatic carriers: ↑ MD in posterior parietal & medial frontal regions
Li et al, 2015	APP, PS1	30 (10; 20)	Symptomatic 46.5 (9.3); Pre-symptomatic 42.7 (8.4); Non-carriers 48.4 (15.1)	TBSS	3	0, 1000	1 × 1 × 1	30	1	Cingulum, superior longitudinal fasciculus, inferior longitudinal fasciculus	• Pre-symptomatic carriers vs controls: ↑ MD left inferior longitudinal fasciculus, left forceps major, right & left superior longitudinal fasiculus, left cingulum • Symptomatic & pre-symptomatic vs controls: pattern as above, differences ↑
Parra et al, 2015.	PS1	58 (22; 14)	Symptomatic 47.5 (6.4); Pre-symptomatic 35.1 (5.5); Non-carriers 39.3 (8.3)	VBA	1.5	0, 1000	1.72 × 1.72 × 3	12	1	Parahippocampal cingulum, genu & splenium of corpus callosum, frontal white matter, parahippocampal cingulum, centrum semiovale	• Pre-symptomatic carriers vs controls: no significant differences • Symptomatic carriers vs controls: ↑ MD in parahippocampal cingulum, left splenium, genu bilaterally, left inferolateral frontal white matter
Ringman et al, 2007	APP, PS1	20 (12; 8)	Symptomatic [age not reported]; Pre-symptomatic 32 (6.4); Non-carriers 36 (6.2)	VBA	1.5	0, 1000	3 × 3 × 3	6	1	Genu & splenium of corpus callosum, frontal white matter, fornix, cingulum, perforant path, corticopsinal tract, whole brain	• Pre-symptomatic carriers vs controls: ↓ FA fornix & frontal white matter • Symptomatic & pre-symptomatic carriers vs controls: ↓ mean FA whole brain, ↓ FA left frontal white matter, right & left perforant path
Ryan et al, 2013	PS1	40 (20; 20)	Symptomatic 49.0 (9.4); Presymptomatic 37.8 (4.7); Non-carriers 44.3 (12.7)	TBSS	3	1000	1.1 × 1.1 × 1.1	64	1	Fornix, cingulum, corpus callosum	• Pre-symptomatic carriers vs. controls: ↓ MD & RD in right cingulum • Symptomatic carriers vs controls: ↓ FA, ↑ MD & LD in all tracts
Sanchez-Valle et al, 2016	PS1	36 (22; 14)	Symptomatic 46.63 (9.1); Pre-symptomatic 39.2 (10.4); Non-carriers 39.0 (9.5)	VBA	3	0	2 × 2 × 2	30	1	Whole brain	• Pre-symptomatic carriers vs controls: no significant differences • Symptomatic carriers vs controls: ↓ FA with ↑ relative age ratio in genus & body of corpus callosum & corona radiate; ↑ MD, LD, RD with ↑ relative age ratio in splenium

Please note that we report findings from the most rigorous analyses conducted by studies, including models controlling for multiple comparisons. When not otherwise reported, NEX was assumed to be 1. Acronyms: dMRI = diffusion Magnetic Resonance Imaging; PS1 = Presinillin 1; APP = Amyloid Precursor Protein; TBSS = Tract-Based Spatial Statistics; VBA = Voxel-Based Analysis; ROI = Region of Interest; FA = Fractional Anisotropy; MD = Mean Diffusivity; RD = Radial Diffusivity; LD = Longitudinal Diffusivity.

### 3.7 Studies of white matter and AD risk loci from GWAS

Three studies correlated white matter metrics with AD risk loci identified through GWAS. One was cross-sectional and two were case/control studies. They all included healthy participants (Mean age range 23.6–72.7; N range 197–645). Two studies used an ROI approach, one used VBA.

Braskie et al. imaged healthy young adults and found that each C allele copy of the *CLU* allele was associated with lower FA in the splenium of the corpus callosum, the fornix, cingulum, and superior and inferior longitudinal fasciculi bilaterally (Braskie et al., 2011). The Lothian Birth cohort study identified lower FA associated with different length genotypes of the poly-T repeat in TOMM40. Shorter genotypes were significantly associated with lower FA in the right rostral cingulum and left ventral cingulum. This effect was independent of *APOE* genotype (Lyall et al., 2014). Foley et al used an Alzheimer’s polygenic score, the weighted sum of the risk loci from GWAS, as a continuous variable. They identified an association between increased AD polygenic score and decreased FA in the right cingulum in young adults (Foley et al., 2016).

Elliot et al undertook a GWAS of brain imaging phenotypes in the UK Biobank cohort (Elliott et al., 2018). They used imaging data from around 15,000 participants. All results are available on the Oxford Brain Imaging Genetics (BIG) web browser (http://big.stats.ox.ac.uk/). The BIG website can be browsed associations by phenotype, gene or SNP. We explored the associations between AD risk loci identified in the Kunkle at al GWAS (Kunkle et al., 2019) and FA/MD derived from TBSS in UK Biobank. A table summarising these results is provided in the Supplementary Materials. Broadly, the results corroborate the findings of other studies included in this review. APOE and CR1 showed particular evidence of association with reduced fractional anisotropy and increased mean diffusivity. However, these results are not corrected for multiple comparisons.

### 3.8 Study quality overview

Most studies scored highly on the NOS. Generally, the comparability of the groups was clearly explained. As the exposure was gene status, there was little possibility of ascertainment bias. Some studies had one point deducted for failing to describe the selection of study groups, particularly of control subjects. The outcomes of interest (white matter metrics) were defined, although the methodology employed to measure these was variable. It was often difficult to assess the quality of the diffusion methodology, as authors often did not provide sufficient information. Most studies gave some details of their pre-processing, although one acknowledged they had not corrected for Gibbs ringing, a common artefact (Gibbs, 1898). The papers generally did not give details of their model estimation technique (for example nonlinear least squares (NLLS), weighted linear least squares (WLLS) or ordinary least squares (OLS)), which can lead to different outcomes (Koay et al., 2006). The majority of studies, 27 of 37, used TBSS or VBA. Of those papers that used tractography, only some described or referenced the specific methods (such as deterministic or probabilistic).

## 4 Discussion

This review establishes that the literature reports AD genetic risk is related to altered white matter microstructure, as indexed by increased diffusivity and decreased anisotropy. By synthesising results across studies, this review demonstrates that AD risk genes were associated with widespread white matter changes, rather than discrete microstructural abnormalities in medial temporal structures such as the fornix. This review also found evidence of changes related to AD risk even in studies of young, healthy adults.

### 4.1 White matter changes associated with AD risk genes

AD genetic risk is associated with reduced anisotropy and increased diffusivity across the brain, most notably in temporal and frontal lobes, cingulum and corpus callosum. Table 3 contains a summary of the five tracts that were implicated in the most studies (tract images were generated using FiberNavigator (​Chamberland et al., 2014​) and TractSeg (​Wasstheral et al., 2018​)). Although some studies reported no differences between pre-symptomatic gene carriers and non-carriers, many of these studies were limited by small sample sizes. Differences between symptomatic carriers and non-carriers frequently paralleled the differences between pre-symptomatic carriers and non-carriers, but in the pre-symptomatic group often fewer regions reached statistical significance or effect sizes were smaller.

**Table 3:** Summary of key findings for the most commonly implicated tracts by gene risk (APOE or FAD).

Tract	Diagram	Summary of APOE Findings	Summary of FAD Findings
*Corpus Callosum:* Connects the left and right cerebral hemispheres		*E4 carriers* vs*. non-carriers:* ↓FA (Persson et al., 2006, Newlander et al., 2014, Heise et al, 2010, Cavedo et al., 2017, Zhang et al., 2015, Ryan et al., 2011, Slattery et al., 2017)*‘*↑MD (Heise et al, 2010, Cavedo et al., 2017, Cai et al., 2017, Zhang et al., 2015) ↑RD (Cavedo et al., 2017, Westlye et al., 2012)	*Pre-symptomatic carriers* vs*. controls:* ↓FA (Sanchez-Valle et al, 2016) Symptomatic carriers vs. controls: ↑MD (Sanchez-Valle et al., 2016, Parra et al., 2015) ↑LD (Sanchez-Valle et al, 2016) ↑RD (Sanchez-Valle et al, 2016)
*Cingulum:* *Connects the temporal and frontal lobes, cingulate and medial gyri of frontal, occipital, parietal and temporal lobes*		*E4 carriers* vs*. controls:* ↓FA (Lyall et al, 2014, Bagepally et al, 2012, Heise et al, 2010, Cavedo et al., 2017, Nierenberg et al., 2005) ↑MD (Li et al, 2012, Zhang et al., 2015, Cavedo et al., 2017, Adluru et al., 2014) ↑RD (Cavedo et al., 2017, Nierenberg et al., 2005)	*Pre-symptomatic carriers* vs*. controls:* ↓MD (Ryan et al, 2013) ↑MD (Li et al, 2012) ↓LD (Ryan et al, 2013) Symptomatic carriers vs. controls: ↓FA (Ryan et al, 2013) ↑MD (Ryan et al., 2013, Parra et al., 2015, Li et al, 2012) ↑LD (Ryan et al, 2013)
*Inferior Occipito-Frontal Fascicle:* Connects the medial temporal *lobe and the inferior frontal lobe*		*E4 carriers* vs*. non-carriers:* ↓FA (Persson et al., 2006, Cavedo et al., 2017) ↑MD (Operto et al, 2018) *↑LD (*Operto et al, 2018*)* ↑RD (Cavedo et al., 2017, Westlye et al., 2012, Operto et al., 2018)	No significant findings
*Superior Longitudinal Fascicle:* Connects the frontal, parietal, occipital and temporal lobes		*E4 carriers* vs *non-carriers:* ↓FA (Heise et al, 2010) ↑MD (Adluru et al., 2014, Operto et al., 2018, Cavedo et al., 2017, Heise et al, 2010) ↑RD (Operto et al., 2018, Westlye et al., 2012) ↑LD (Operto et al, 2018)	*Pre-symptomatic carriers* vs *non-carriers:* ↑MD (Li et al, 2012) Symptomatic carriers vs non-carriers: ↑MD (Li et al, 2012)
*Inferior Longitudinal Fascicle:* Connects the occipital pole and temporal pole		*E4 carriers* vs *non-carriers:* ↓FA (Lyall et al., 2014, Cavedo et al., 2017) ↑MD (Operto et al, 2018) ↑RD (Operto et al., 2018, Dowell et al., 2013, Cavedo et al., 2017) ↑LD (Operto et al, 2018)	*Pre-symptomatic carriers* vs *non-carriers:* ↑MD (Parra et al, 2015, Li et al, 2012) Symptomatic carriers vs non-carriers: ↑MD (Li et al, 2012)

Acronyms: E4 = APOE Epsilon 4; FAD = Familial Alzheimer’s Disease; FA = Fractional Anisotropy; MD = Mean Diffusivity; RD = Radial Diffusivity; LD = Longitudinal Diffusivity. Tract images were generated using FiberNavigator (Chamberland et al., 2014) and TractSeg (Wasserthal et al., 2018).

The literature included in this review reported diffuse changes in white matter signal and global structural connectivity. These reflect the changes across regions and hemispheres that underpin emergent AD. There was significant overlap between the regions implicated by studies of APOE, autosomal-dominant AD genes and GWAS loci. This suggests that although these genes are involved in different biological processes, these pathways may converge on a common final pathway resulting in a corresponding pattern of neurodegeneration. This is in keeping with the literature on AD pathology (Naj and Schellenberg, 2017). However, there was no evidence that microstructural changes were related to any individual microstructure component, as abnormalities were evident across white matter metrics.

### 4.2 Methodological considerations

**Fig. 2:**
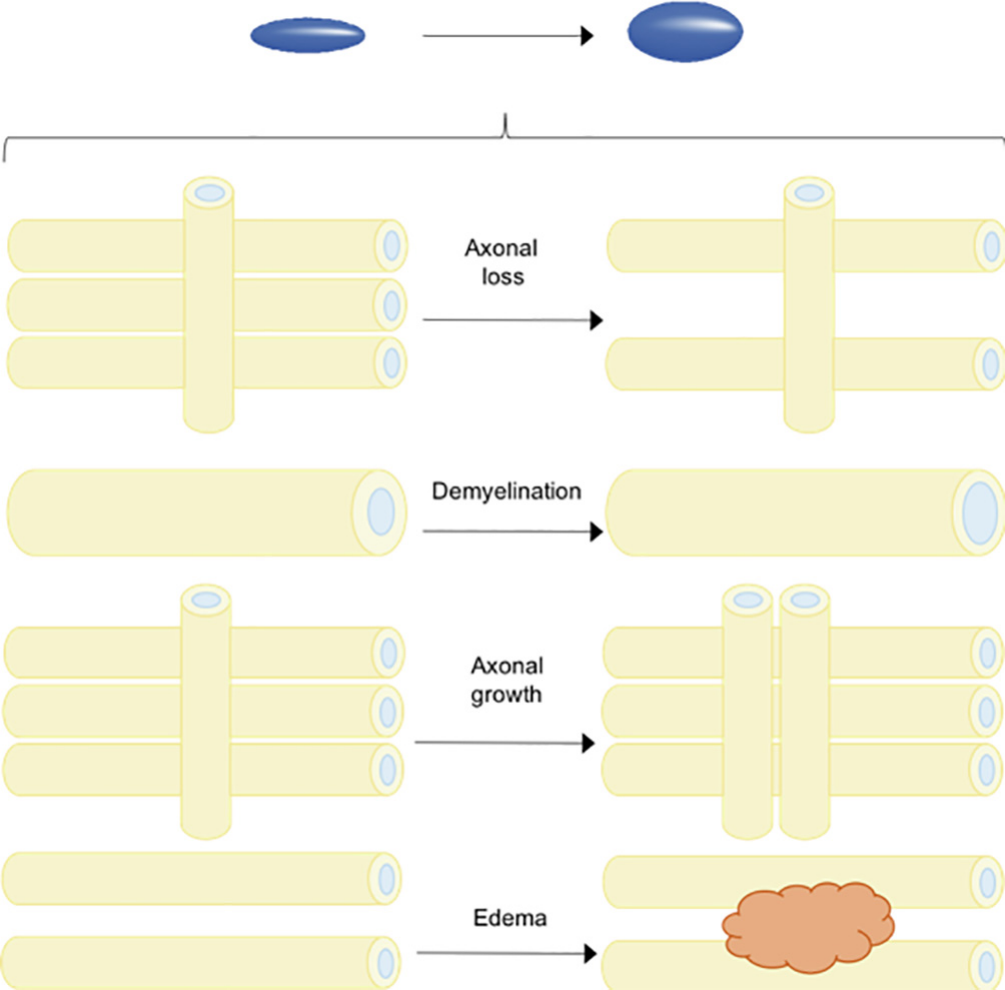
The change in the diffusion signal (isotropic to anisotropic diffusion) can result from multiple different pathologies. States that can produce the same signal change include axonal loss, demyelination, axonal growth or edema.

The field has some key limitations (Jones and Cercignani, 2010, Jones et al., 2013). Firstly, water diffusion is not a direct measure of neuroanatomy. Secondly, dMRI is an intrinsically noise-sensitive and low-resolution technique (Jones, Knösche and Turner, 2013). Several dMRI models assume fibre bundles to run parallel in a tract. However, fibres cross perpendicularly within voxels in many brain regions, which reduces the FA. The percentage of voxels containing crossing fibres is estimated to be ∼ 90% (Jeurissen et al., 2013). It is also difficult to separate tracts that are closely aligned and then diverge (Tournier et al., 2011). DTI also demonstrates ‘degeneracy’: the same change in the diffusion tensor can be explained by multiple processes e.g., differently oriented fibre populations (‘crossing fibres’), or the ratio intra/extra-axonal space (see Fig. 2). Therefore dMRI is sensitive but lacks specificity (Jelescu et al., 2016a) and cannot provide an interpretable marker other than a vague concept of ‘tissue integrity’ (Wheeler-Kingshott and Cercignani, 2009).

### 4.3 Interpretation of dMRI signal change in AD

In additional to neurodegeneration, a number of different pathological processes can result in the same changes in diffusion signals. However, the presence of abnormal dMRI measures in AD correlates with other AD biomarkers, such as amyloid PET (Kantarci et al., 2014), CSF amyloid-beta and phosphorylated tau (Amlien et al., 2013, Gold et al., 2014, Li et al., 2015). Among those with AD, lower Mini-Mental State (MMSE) scores are associated with a greater effect size for FA in several brain areas, particularly the parietal region.

There is still much debate about the pathophysiology underpinning white matter changes in AD. For example, it is not clear whether white matter alterations are related to, or independent of, gray matter degeneration in AD. One hypothesis is that changes in white matter microstructure result from Wallerian degeneration (Coleman, 2005). According to this hypothesis, patterns of white matter alterations should correspond to grey matter pathology, occurring first in the hippocampal and entorhinal areas, before extending to wider temporal and parietal regions (Braak & Braak, 1997). Conversely, the theory of retrogenesis suggests that those tracts which are last to myelinate are the first to degenerate (Reisberg et al., 2002, Bartzokis, 2004). In this case, late-myelinating tracts would be affected first. It was striking that in the results of our systematic review there were no longitudinal dMRI studies comparing those at high and low genetic risk at different time points. Such debates cannot be resolved without serial imaging to assess dynamic changes in white matter signal.

Caution is required when interpreting diffusion metrics in AD. Some AD dMRI studies have concluded their findings showed disruption of myelin rather than axon damage based on the effect on LD relative to RD (Operto et al. 2018). Indeed, authors of ex-vivo studies in rats (Nevo et al., 2001) and mice (Song et al., 2002) as well as a small study of cervical spondylosis patients (Ries et al., 2000) have suggested that a decrease in LD and increased in RD could potentially be used to differentiate demyelination from axonal injury. However, it may not be safe to generalize findings from controlled animal experiments and spinal cord studies to human brain, which has complex white matter architecture. Microstructural dMRI models (Assaf and Basser, 2005, Panagiotaki et al., 2012, Zhang et al., 2012), which aim to be more specific than dMRI by describing the signal as arising from a sum of tissue compartments, hold great promise, but the nonlinear fitting suffers from poor precision (Jelescu et al., 2016a). Furthermore, microstructural dMRI models do not account for water in myelin because it cannot be detected with common dMRI acquisitions. Measuring myelin content is relevant for monitoring pathologies where demyelination, dysmyelination and remyelination are implicated. Thus, despite dMRI signals being modulated by changes in myelin content through changes in intra/extra-axonal space (Jelescu et al., 2016b), it can only reveal 'part of the picture'.

### 4.4 Strengths and limitations of this review

We followed PRISMA guidelines and used a comprehensive systematic search strategy to avoid missing relevant studies. We did not narrow our eligibility criteria to studies using particular research designs (e.g. case/control studies), samples (e.g. only clinical or healthy) or only young or older participants. We also included studies using any dMRI technique (e.g. TBSS, VBA or tractography-based ROI) or analysis (e.g. structural connectivity) to enhance our ability to evaluate how AD genetic risk is manifest in white matter. Unfortunately, any eligible studies in non-English language journals would have been overlooked. The methodology was heterogeneous. Even when the same techniques are applied there can be differences between scanners (Vollmar et al., 2010), and although some standardisation exists for DTI acquisition, other designs are largely ad hoc and can vary between centres. This meant that we were unable to perform a *meta*-analysis, could not establish the magnitude of effect sizes or assess for publication bias. The studies included in this review had a number of limitations. Some of the studies were probably underpowered. Authors often failed to describe sample ascertainment, making it more difficult to contextualise their results. The majority of studies included used either TBSS or VBA, which have a number of limitations, such as the requirement for spatial smoothing (Jones et al., 2005, Jones and Cercignani, 2010, Edden and Jones, 2011).

### 4.5 Potential clinical applications

As this review demonstrates, there is evidence that dMRI markers can detect changes in white matter microstructure in those with increased genetic risk of AD. The evidence suggests that some white matter tracts may be more sensitive than others, offering a possible marker of incipient disease. dMRI may also prove to be a useful tool for monitoring disease progression. However, dMRI presents a number of methodological challenges, and the biological changes that underpin alterations in dMRI signal are uncertain. However, with continuous improvements in imaging technology (McNab et al., 2013, Jones et al., 2018), and biophyiscal modeling (Novikov, Kiselev and Jespersen, 2018), we are likely to deepen our understanding of those biological underpinnings. Conventional T1- and T2- weighted images give established diagnostic markers and are widely used in clinical practice (Frisoni et al., 2010). The utility of dMRI as an adjunct to traditional structural assessment is as yet unproven. Beyond that, there are also practical challenges, such as the length of acquisition protocols, and a lack of standardisation of models, acquisition and analysis.

## 5 Conclusions

Despite some methodological limitations, the majority of the studies presented in this review demonstrate significant associations between AD genetic risk and diffusivity in white matter tracts. Specifically, lower FA and increased MD, RD and LD were found in a number of white matter tracts. This review emphasises the need for longitudinal studies of AD genetic risk to fully characterise white matter changes related to neurodegeneration across the lifespan. It is probable that very early pathology will be more amenable to therapeutic intervention. Therefore, early detection and pre-symptomatic treatment are vital. As acquisition and analysis techniques develop, dMRI is able to provide increasingly detailed information about the structure of white matter and brain connections and may develop useful biomarkers for AD pathology in future.

## CRediT authorship contribution statement

**Judith R. Harrison:** Conceptualization, Formal analysis, Funding acquisition, Methodology, Project administration, Supervision, Writing - review & editing. **Sanchita Bhatia:** Data curation, Investigation, Methodology, Project administration, Writing - review & editing. **Zhao Xuan Tan:** Investigation, Writing - review & editing. **Anastasia Mirza-Davies:** Investigation, Writing - review & editing. **Hannah Benkert:** Investigation, Writing - review & editing. **Chantal M.W. Tax:** Visualization, Methodology. **Derek K. Jones:** Supervision, Methodology.

## Declaration of Competing Interest

The authors declare that they have no known competing financial interests or personal relationships that could have appeared to influence the work reported in this paper.
