# Supplementary material for: Imaging Alzheimer's genetic risk using diffusion MRI: A systematic review
Source: Neuroimage Clin. 2020 Jul 22;27:102359. doi: 10.1016/j.nicl.2020.102359 (PMC7399253; doi:10.1016/j.nicl.2020.102359)
Supplement: Supplementary data 1 [file mmc1.docx]

**Supplementary Material**

Table 1: Literature Search Methodology

| Search Terms: | Inclusion Criteria: | Exp. Alzheimer*  Alzheimer’s Disease  Mild Cognitive Impairment, MCI  Familial Alzheimer’s Disease (autosomal-dominant AD)  Apolipoprotein E type 4, ApoE4  Presenilin 1 and 2 (*PS1* and *PS2*)  Amyloid beta-Protein Precursor, (APP)  Cognitive Performance  Cognitive Disorder  Cognitive Dysfunction  Dementia |
| --- | --- | --- |
|  | AND | Exp. Diffusion Tensor Imaging*  Diffusion Tensor Imaging  DTI  Diffusion Magnetic Resonance Imaging  dMRI  White Matter Microstructure  White Matter  White Matter Integrity  Connectome  Connectomics |
|  | Selection: | English Language  Humans  Year: 2000 – 2019 |
|  | Exclusion Criteria: | Dementia (unspecified)  Vascular dementia  Lewy Body dementia  Other dementia  Huntington’s  Unspecified memory decline  Other Neuropsychiatric disorder  Cognitive function – unrelated to Alzheimer’s pathology  Other Imaging modalities  Acquisition method – FMRI, volumetric studies |

Table 2. List of data extracted

| Paper Title |
| --- |
| Author |
| Year |
| Population Group (i.e. MCI, AD patients, APOE carriers) |
| Abstract |
| N |
| Participant Age |
| Gene type (e.g. APOE, polygenic, familial, etc) |
| Study design (e.g. Case-control, etc) |
| Quality Assessment Score (e.g. Newcastle Ottawa Score) |
| Country of Origin |
| Diffusion Method (i.e. ROI, TBSS) |
| Field Strength (T) |
| B value (s/mm2) |
| Acquisition Voxel Size |
| Number of Directions |
| NEX |
| Pre-processing |
| Model Estimation |
| Anisotropy Measure |
| Diffusivity Measure |
| Other Metric Brain Region(s) |
| AD Associated Symptoms |
| Key Results |
| Methods Text |

Table 3. PRISMA Checklist


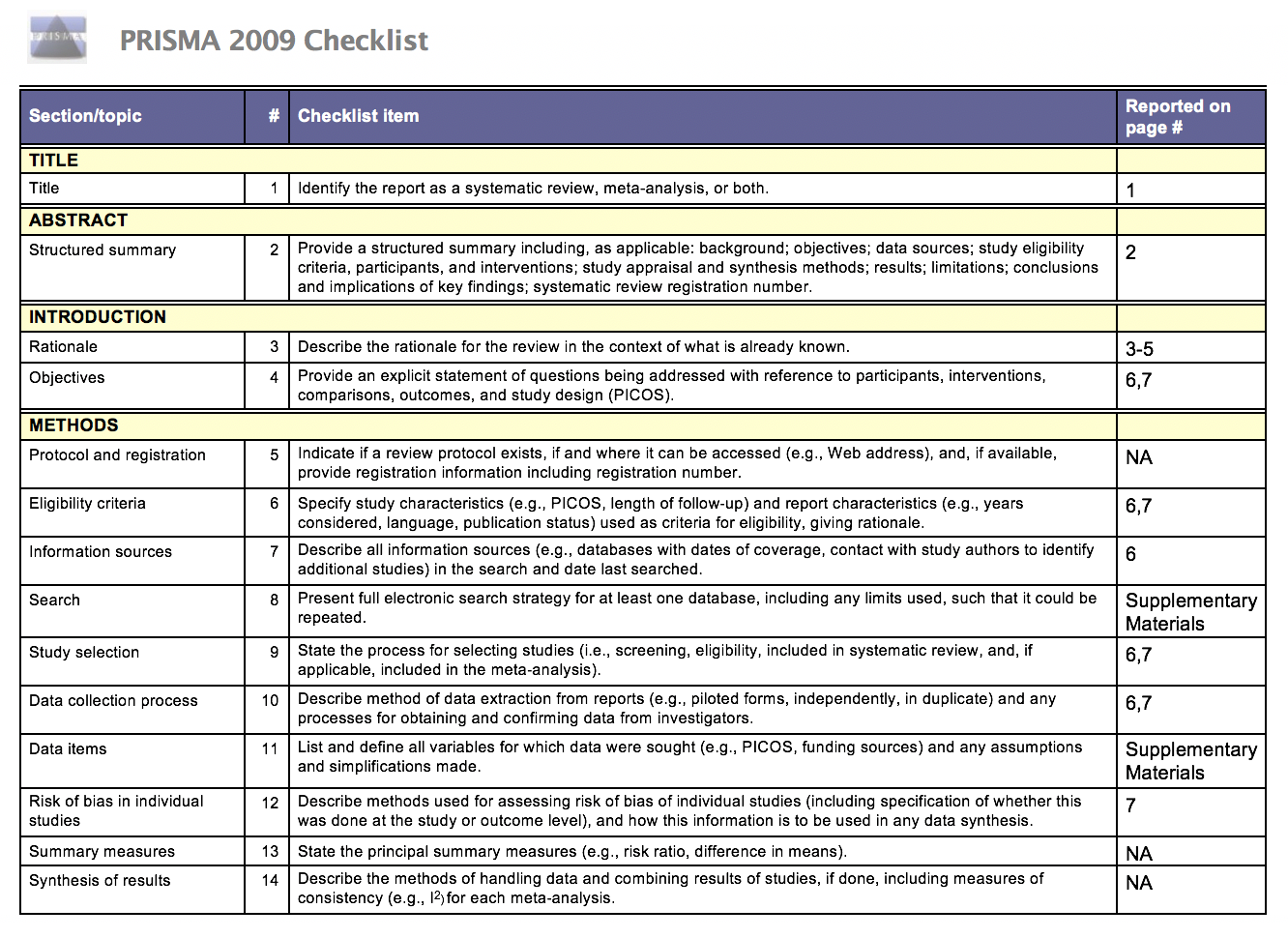

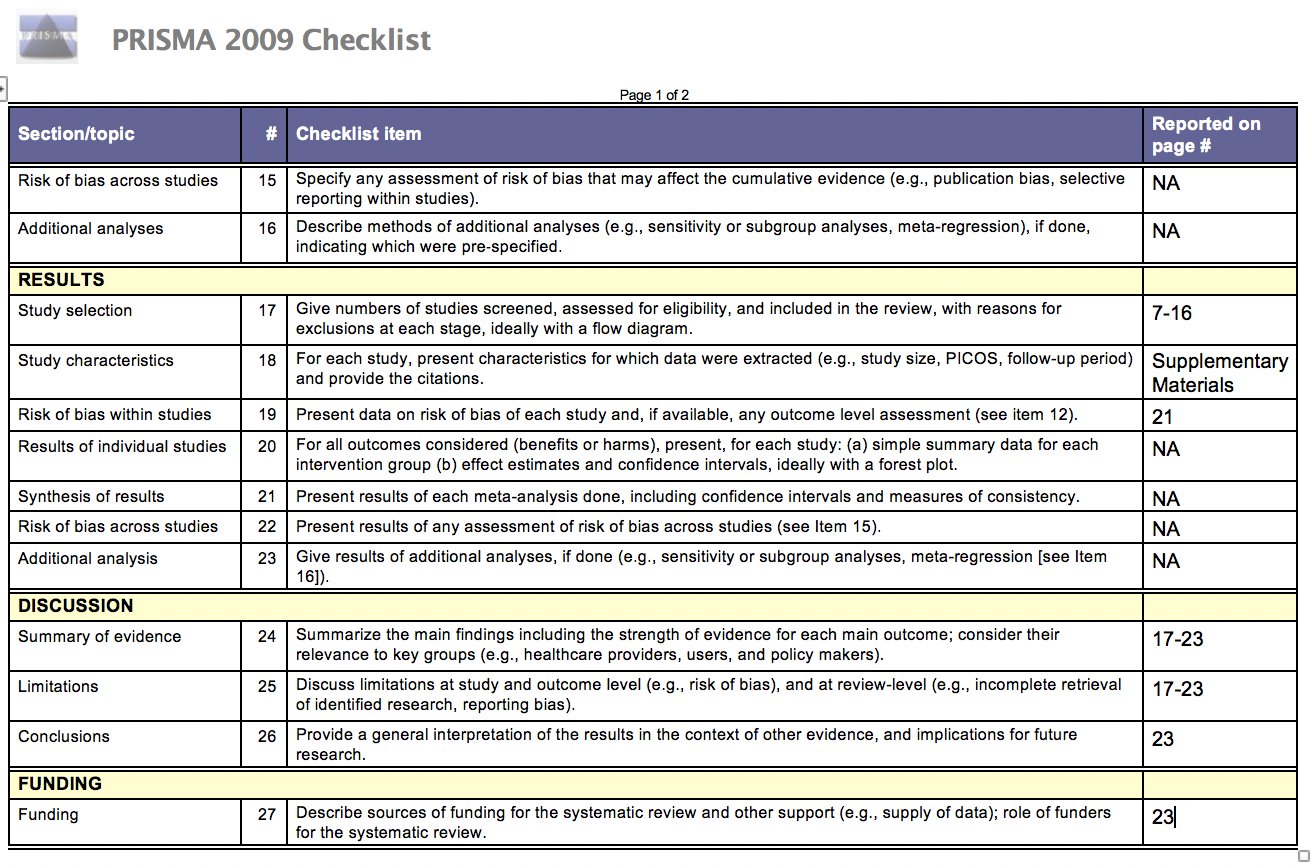


Table 4. Summary of UK Biobank genetic imaging findings
